# Actuating Bimorph Microstructures with Magnetron-Sputtered Ti-Ni-Cu Shape Memory Alloy Films

**DOI:** 10.3390/nano12234207

**Published:** 2022-11-26

**Authors:** Vlad Bolocan, Dragos Valsan, Aurel Ercuta, Corneliu-Marius Craciunescu

**Affiliations:** 1 Department of Materials and Manufacturing Engineering, Faculty of Mechanical Engineering, Politehnica University Timisoara, P-ta Victoriei nr., 30332 Timisoara, Romania; 2 Technical Sciences Academy of Romania, Bulevardul Dacia 26, 030167 Bucharest, Romania

**Keywords:** shape-memory alloys film, martensitic phase transformation, Ti-Ni-Cu system, DC magnetron sputtered, actuation, modeling

## Abstract

The generation of microactuation using narrow thermal hysteresis Ti-Ni-Cu shape-memory alloy films deposited on non-metallic substrates as the active element is studied based on a model previously developed for Ni-Ti/Si bimorphs. To this end, the compositional range in which the B2 (monoclinic) → B19 (orthorhombic) martensitic phase transformation occurs was considered, and films were deposited by magnetron sputtering on heated Si and Kapton substrates. Ultra-fine grains were observed for the 550 °C deposition temperature. The selected composition was close to Ti_50_Ni_35_Cu_15_, so the narrowing of the thermal hysteresis is not associated with a significant reduction in shape recovery capability. The microstructure and composition of the target materials and as-deposited films used in our experiments were characterized by X-ray diffraction and scanning electron microscopy, whereas the temperature dependence of the volume fraction of the martensite phase was derived using differential scanning calorimetry records for the target materials and from the temperature dependence of the electrical resistance data for the films. An original model was used to predict the actuation of cantilever-type bimorphs with Kapton and Si substrates.

## 1. Introduction

Shape-memory Alloys (SMAs) are materials that exhibit some peculiar properties, such as thermal and/or magnetic shape recovery, superelasticity, high mechanical damping, and even biocompatibility for some compositions [1] that make these alloys of high interest for medical [2], as well as for non-medical applications [3]. The shape-memory effect, materialized through the capacity to recover the shape following a plastic deformation, also makes them useful for manufacturing actuators that can be used in microelectromechanical systems.

In transferring the shape-memory alloy properties to lower dimensional ranges actuation, several issues need to be considered, such as the generation of actuation on heating and cooling using bimorph structures, the composition of the shape-memory alloys and the substrates used to generate the bimorphs, and the effect of the deposition temperatures on the resulting actuation.

The aim of the paper is to extend the simulations based on the model developed for Ni-Ti shape-memory alloys deposited on Si substrates (or other strong Young modulus and low-CTE materials to polymeric substrates (low Young’s moduli and high CTE)) and to experimentally analyze the microactuation of Ti-Ni-Cu shape-memory alloys with lower hysteresis deposited on such substrates.

The particular behavior of SMAs is based on the martensitic phase transformation (MPT) that, for most SMAs, occurs in the vicinity of room temperature (RT) [4,5]. The structural transformation from the low-temperature stable phase (martensite) to the high-temperature stable phase (austenite) is both thermoelastic and reversible and can be used for actuation, especially in applications that require low frequency and high actuation output [6]. Apart from routine metallurgical procedures, more recently, SMAs were developed as rapidly solidified ribbons and thin films [7,8], and thus, successful efforts were made in microactuator design [9,10,11] and their implementation in building microelectromechanical systems (MEMS) [12,13].

The reversible martensitic phase transformation, responsible for the shape recovery after heating, is more effective in terms of actuation if an elastic element deforms and stores the energy during heating (when the martensite transforms into austenite) and deforms the SMA during cooling (when the stiffer austenite transforms into the softer martensite phase) [14].

The deposition of SMA films is used for sensor and actuator fabrication (e.g., [15]) and for new or optimized film composition based on combinatorial exploration [16]. The advantage of film sputtering on heated substrates mainly consists of the fact that the bonding between the film and the substrate is formed at the deposition temperature. Thus, upon cooling from the deposition temperature, thermoelastic stress develops in the bimorph formed at high temperatures. A similar case is when the film is deposited at room temperature (RT) and is further annealed for a full crystallization at a high temperature, usually in the 500–650 °C range.

Depending on the composition of the SMA, one or more phase transformations can occur, and—since they imply a structural change—they are also associated with a change in SMA film properties, which is reflected in the thermoelastic stress and implicitly in the actuation of the bimorph-type cantilevers. The typical phase transformations in solution-treated NiTi-based shape-memory alloys usually follow a B2 (cubic) → B19’ (monoclinic) sequence. When additional alloying elements are added, changing the equiatomic composition, or in aged NiTi alloys or aged nitinol, intermediary phases (e.g., orthorhombic-B19 or rhombohedral-R-phases) can occur. Compared to Nitinol, Ti-Ni-Cu alloys are particular shape-memory alloys considering that their sequence of phase transformation is influenced by the amount of Ni that is substituted by Cu. Depending on the composition, NiTiCu shape-memory alloys undergo either a B2 → B19’ (for lower Cu content) sequence or B2 → B19 → B19’ (for higher Cu content) sequence, where lower thermal hysteresis may be obtained [17], with the positive influence of the actuation frequency. B19 orthorhombic martensite (B19) was observed by Shugo et al. [18] when Cu replaced more than 10% Ni, which was confirmed by Tadaki et al. [19].

The change in the sequence of transformation is also associated with changes in thermal and mechanical behavior. Based on an extensive compositional study, Nam et al. [20] observed the influence of Cu content for Ti_50_Ni_(50−x)_Cu_x_ alloys and showed that increasing the Cu content in the B2 → B19 compositional range leads to a reduction in the thermal hysteresis and an increase in the B2 → B19 transformation temperatures, while the B19 → B19’ transformation shifts toward lower temperatures. Thus, the replacement of Ni by Cu leads to a decrease in the phase transformation hysteresis from about 20 K at 5% Cu for the B2 ↔ B19’ transformation to about 4 K at 20% Cu for the B2 ↔ B19 transformation.

On the other hand, based on the experiments performed by Li et al. [21], Liu and Hao [22] assessed the Ti-Ni-Cu thermal and mechanical properties in the range of 0–7% Cu, showing that the Young modulus of the austenite phase changed from 31 GPa for the Ti-50.8 Ni to about 35 GPa for Ti-40.8 Ni-10 Cu, while the corresponding Young’s modulus in the martensitic phase decreased from 28 GPa to 26.7 GPa for the same compositional range.

One of the issues with increasing the Cu content is caused by the poor workability observed for Cu contents exceeding 10 at.% Cu. However, it was reported the brittleness of the Ti-Ni-Cu alloys could be avoided through rapid solidification, as is the case for melt-spun ribbons and for films deposited by magnetron sputtering.

Over time, several other substrates have been explored to assess the possible use in various applications based on shape-memory alloy films deposited on substrates, substrates that can be dissolved following the deposition, or out of which the film may be removed, leading to freestanding films. Common substrates used for the deposition are glass [23], silicon [24], and metal (e.g., on Mo [25] or 12 µm Cu substrate [26]); however, free-standing films have also been manufactured [27]. Silicone has the advantage that it can be manufactured using well-known microfabrication technologies. Glass, on the other hand, is readily available and less expensive. Although there are excellent possibilities for manufacturing and generating reproducible results, one of the disadvantages of the two mentioned substrates is that they are brittle and not always easy to post-process in the desired form. Thus, the use of polymeric materials can be a solution to accelerate the development of microactuators by using lower-cost substrates that can be manufactured in a more cost-efficient way.

In most cases, the shape-memory alloy films deposited at room temperature need to be annealed in order to show the shape-memory effect. In these cases, an annealing temperature in the range of 600 °C or higher may be needed. For such annealing temperatures, it is difficult to find a wide palette of adequate polymeric substrates. Another way is to deposit the films on heated substrates, a technique requiring lower substrate heating temperatures. It was shown that for NiTi films deposited on Si substrates, the crystallization starts at temperatures above 250–300 °C [28], whereas, for Ni-Mn-Ga freestanding films, the occurrence of the ferromagnetic and shape-memory properties sequence onsets during crystallization by heating above 350 °C and 600 °C, respectively [29]. The deposition temperature required for actuation is influenced by the composition. For example, it was shown that Ti-Ta films could show shape-memory properties by deposition of unheated substrates [30].

One possibility is to use polymeric substrates that can be easily manufactured prior to or after the deposition of the SMA. Ishida et al. [31] were successful in depositing crystalline 8 µm films out of Ti, Ni, and Cu elemental targets on heated polyimide substrates using a carrousel-type magnetron-sputtering system, thus opening the path for the use of such substrates for manufacturing shape-memory alloy thin film actuators. The films peeled off out of glass substrates also showed the B2-B19 reversible transformation. Hou et al. [32] pointed out the importance of the substrate temperature and the fact that this can be challenging for polymeric substrates with shape-memory effect found for 3 μm TiNi sputtered at 703 K on 7.6 μm thick Kapton^®^ polyimide substrate. Jayachandran et al. [33] deposited 1.75 μm NiTi and Cu-based films on a 50 μm pre-strained Kapton polyimide sheet using a flash evaporation technique. Pre-straining was mentioned as a step in generating the microactuators.

Kumar et al. [34] deposited Ti_55_Ni_45_ films with a thickness of 1 µm on heated (100) Si substrates and found that crystallization occurred after annealing at 500 °C for the films deposited at 300 °C that were amorphous even for deposition at 400 °C (with the hardness in the 12 GPa range for the films annealed at 600 °C and the elastic modulus in the range of 180 GPa after annealing). Mohri et al. [35] deposited 1µm Ni-rich NiTi films (300 W DC magnetron sputtering for 1 h at RT) on 25 µm-thick Kapton 100 HN and found that after annealing at 450 °C for 30 min that a superelastic effect was present in the bimorph. In terms of how the film crystallinity influences the SMAs functionality, it was shown by Rumpf et al. [29] that in order to observe the MPT in Ni-Mn-Ga ferromagnetic films, these alloys need to have a certain degree of crystallinity higher than that required for magnetic properties. Even bulk materials, such as melt-spun ribbons [36] and melt-suctioned bars [37], or materials submitted to severe plastic deformation [38] can have adjusted crystallinity. In such cases, MPT is reflected in broader DSC peaks, of lower intensity, for the partially crystalline state [39].

The present work extends our simulations based on the model proposed in [40] for a Ti-Ni/Si bimorph cantilever in order to predict the actuation behavior of such a device now using narrow thermal hysteresis Ti-Ni-Cu films and explore the possibility of using Kapton as a substrate. The model is based on Timoshenko’s theory of the bimetal thermostat [41], into which the temperature-dependent thermoelastic properties of the SMA film, here represented by the coefficient of thermal expansion α*_f_*(*θ*), and Young’s modulus E*_f_* (*θ*), are incorporated; during the MPT, these quantities will evolve between their values in the two stable phases (M-martensite and A-austenite), i.e., *α*_M_ and *α*_A_, as well as *E*_M_ and *E*_A_, respectively. 

## 2. Materials and Methods

Ni-Ti and Ti-Ni-Cu targets were used for depositing films on Si, DuPont™ Kapton^®^ HN polyimide and on glass substrates, resulting in film/substrate bimorph structures. The substrates were placed on a heating plate during the entire deposition that was made with the following parameters: 2 × 10^−6^ Torr preliminary vacuum, 10 mTorr Ar pressure and 100 W power, using a magnetron sputtering system AJA Orion 8 (AJA International, Scituate, MA, USA). The temperature of the substrate was controlled via quartz lamps placed behind the Inconel plate.

Silicon and *DuPont*™ *Kapton*^®^ *HN* (courtesy of DuPont), with a thickness in the range of 100 µm, were used as substrate materials, whereas the thickness of the deposited films (in the range of 0.5–2 µm) was determined by means of a Sloane Dektak II stylus profilometer (Veeco-Sloane, Plainview, NY, USA).

The target and the film composition were analyzed in a TESCAN Vega 3LM electron microscope (TESCAN Brno s.r.o., Brno, Czech Republic) equipped with a Bruker Quantax 200 Energy Dispersive X-ray Spectroscopy (EDX) system with Peltier-cooled XFlash 410 M silicon drift detector. The structure of the film was studied by X-ray diffraction with Panalytical X’Pert Pro equipment (Eindhoven, The Netherlands), using Cu_kα_ radiation (λ = 1.5405 Å).

Isochronal differential scanning calorimetry (DSC) was used to explore the forward and reverse MPT in the target material using Perkin Elmer DSC7 equipment (Waltham, MA, USA) with Pyris software. After removing the heat capacity change during the MPT, and of the baseline, as the mass densities of the two phases are very close (e.g., in Ti_50_Ni_50_, the two densities practically coincide), the profile of the as-corrected DSC device output signal (the heat flow per unit mass and time vs. temperature), w(*θ*), will scale with the rate of the change in the transformed phase volume fraction [42]. Then, during a full heating/cooling scan, the martensite volume fractions along the two branches of the thermal cycle may be expressed as:

(1) $$m_{h}(\theta )=1-\frac{\int_{\theta_{h_{\;1}}}^{\theta }w_{h}\left(\theta '\right)d\theta '}{\int_{\theta_{h_{\;1}}}^{\theta_{h_{\;2}}}w_{h}\left(\theta '\right)d\theta '}\;\theta_{h_{\;1}}\leq \theta_{A_{s}}\leq \theta \leq \theta_{A_{f}}\leq \theta_{h_{\;2}}\;(heating)$$

(2) $$m_{c}(\theta )=\frac{\int_{\theta_{c_{\;1}}}^{\theta }w_{c}\left(\theta '\right)d\theta '}{\int_{\theta_{c_{\;1}}}^{\theta_{c_{\;2}}}w_{c}\left(\theta '\right)d\theta '}\;\theta_{c_{\;1}}\leq \theta_{M_{f}}\leq \theta \leq \theta_{M_{f}}\leq \theta_{c_{\;2}}\;(coolling)$$

which means that analytic expressions may be obtained for m*_h_*(*θ*) and m_c_(*θ*) from numerical integration of w_h_(*θ*) and w_c_(*θ*), followed by the least square fitting procedure based on transition functions.

However, if the above method is successfully applied to bulk samples (such as small chips from the target material), this becomes hardly possible for SMA films with thicknesses in the micrometric range, in which case, other physical methods should be used. In this sense, based on the fact that the electrical resistivity of the SMAs exhibits different values in the two stable phases, our choice was the temperature dependence, ρ(*θ* ), of the electrical resistivity as recorded during a heating/cooling cycle covering the forward and reverse MPT. Using the 4 points in-line method, the resistivity of a 1 μm-thick Ti_52.28_Ni_33.3_Cu_12.29_ film was determined. To this end, a constant current (I = 15 mA) was injected through the outer pair of electrodes (the bias electrodes), and the voltage drop across the 2 mm-long segment between the inner pair of electrodes (the sense electrodes) of the SMA film, say V(*θ*), was recorded as the temperature varied, both during heating and cooling. By controlling the dc current passing through a pair of stacked TEC1-12708 Peltier modules (P&N Tech Co), on whose top surface the bimorph substrate and the hot junction of a K-type thermocouple were placed in thermal contact, the temperature was changed linearly. Let V_th_(*θ*) be the thermocouple output. Signals V_th_(*θ*) and V(*θ*) were measured by means of a 6½ digit precision MA3510 (Picotest Corp) multimeter and were simultaneously recorded to a PC in numeric format (the former signal was converted into °C). As both of these signals took values in the mV range (e.g., during the forward and reverse transformation, V(*θ*) varied from 5.09 to 5.22 mV, while V_th_(*θ*) did not exceed 4 mV), the inevitably present electromagnetic noise caused an unwanted spread (in the tens of nV range) of the measured data; using least squares interpolation, this spread was corrected, and the resulting data were used to calculate the expected evolution of ρ(*θ*), and from here, an analytic form was derived for the temperature dependence, m(*θ*), of the volume fraction of martensite. To this end, a phenomenological electric model was developed under the same assumption as in the case of the SMA target, i.e., the new phase nucleation sites are distributed at random. Again, function m(*θ*) will be derived from averaging between two bounding approximations, i.e., *serial* and *parallel*; now, these terms refer to the equivalent resistance of the biphasic sample (a rectangular strip of length *l* and uniform cross section *S*), resulting from two single-phase volumes connected (relative to the current direction) either in series, i.e., *V*_M_(*θ*) = *l*_M_(*θ*)∙*S*, and *V*_A_(*θ*) = *l*_A_(*θ*)∙*S*, with *l*_M_(*θ*) + *l*_A_(*θ*) = *l*, or in parallel, i.e., *V*_M_(*θ*) = *l*(*θ*)∙*S*_M_, and *V*_A_(*θ*) = *l*(*θ*)∙*S*_A_, with *S*_M_(*θ*) + *S*_A_(*θ*) = *S*. In addition, as the calculus proves, thermal expansion has a negligible effect on the film geometry within the explored temperature range, and the resistivity scales with the electrical resistance. Correspondingly, the equivalent electrical resistance of the strip, R(*θ*), will take one of the two forms:

(3) $$R_{ser}(\theta )=\frac{\rho_{M}(\theta )l_{M}(\theta )}{S}+\frac{\rho_{A}(\theta )l_{A}(\theta )}{S}$$

or

(4) $$R_{para}(\theta )={\left(\frac{S_{M}(\theta )}{\rho_{M}(\theta )l}+\frac{S_{A}(\theta )}{\rho_{A}(\theta )l}\right)}^{-1}$$

where

(5) $$\rho_{M}(\theta )=\rho_{M}(\theta_{o})\left[1+\gamma_{M}(\theta -\theta_{o})\right]$$

and

(6) $$\rho_{A}(\theta )=\rho_{A}(\theta_{o})\left[1+\gamma_{A}(\theta -\theta_{o})\right]$$

are the linear temperature dependences of the resistivity in the martensite (*M*) and austenite (*A*) stable phases, respectively, within the regions adjacent to the temperature range of the MPT; a reference temperature, conveniently chosen, was denoted by *θ*_o_, whereas γ*_M_* and γ*_A_* are the temperature coefficients of the resistivity in the two phases. Then, from fitting to robust lines, the above linear R(*θ*) or ρ(*θ*) arrays using the fixed reference temperature (*θ*_o_ = 0 °C was chosen), parameters γ*_A_*, γ*_M_*, ρ*_A_*(*θ*_o_), and ρ*_M_*(*θ*_o_) results, and simple calculations lead to the following relations:

(7) $$m_{ser}(\theta )=\frac{\rho (\theta )-\rho_{A}(\theta )}{\rho_{M}(\theta )-\rho_{A}(\theta )}\;(\mathrm{serial})$$

and

(8) $$m_{para}(\theta )=\frac{\frac{1}{\rho (\theta )}-\frac{1}{\rho_{A}(\theta )}}{\frac{1}{\rho_{M}(\theta )}-\frac{1}{\rho_{A}(\theta )}}\;(\mathrm{parallel})$$

between the resistivity and the martensite volume fraction; for simplicity, we shall disregard indices *ser* and *para*, from this point, and will use notation m(*θ*).

Then, by averaging the results of applying Equations (7) and (8) to the ρ(*θ*) data calculated from the as-measured R(*θ*), the numerical values of the martensite volume fraction will be obtained for the two branches (heating and cooling) of the thermal cycle, and again an analytic expression for m(*θ*) will result from the least square fitting procedure based on transition functions.

The thermal evolution of the cantilever deflection was determined in a custom-made system that allowed recording the displacement of the free end of cantilevers as a function of temperature using a laser sensor (OMRON ZX2-LDA, Kyoto, Japan). In both cases, the heating and cooling were performed using Peltier devices.

In order to simulate (in terms of our previously mentioned model) the actuation of our bimorph structure, we shall express the temperature-dependent curvature κ(*θ*) of a cantilever built from a substrate strip of thickness *d_s_*, on which an SMA film of thickness *d_f_* was deposited at temperature *θ*_D_, as:

(9) $$\kappa \text{​}\left(\theta \right)=\frac{6}{d_{f}}\;\frac{\left[\alpha_{s}-\alpha_{f}\text{​}\left(\theta \right)\right]}{3\left(1+\frac{d_{s}}{d_{f}}\right)+\frac{d_{s}E_{s}+d_{f}E_{f}\;\left(\theta \right)\;}{\left(d_{s}+d_{f}\right)E_{s}}\left(\frac{d_{f}}{d_{s}}+\frac{d_{s}^{\;2}E_{f}\;\left(\theta \right)\text{​}}{d_{f}^{\;2}E_{s}}\right)}\;\left(\theta -\theta_{D}\right)$$

Clearly, to render Equation (9) useful for a quantitative description, analytic forms of *α_f_* (*θ*) and *E_f_* (*θ*) are necessary, and for this purpose, the model proposed in Ref. [43] for the thermoelastic behavior of composites consisting of two solid phases, locally arranged in *serial* (isostrain) or *parallel* (isostress) configuration, will be adopted. In these terms, the calculus yields:

(10) $$\begin{array}{l} \alpha_{f\text{​},ser}\text{​​}\left(\theta \right)=m\text{ ​}\left(\theta \right)\alpha_{M}+\left[1-m\text{ ​}\left(\theta \right)\right]\alpha_{A}^{{}^{}}\\ E_{f\text{​},ser}\text{​​}\left(\theta \right)=\frac{E_{M}E_{A}}{E_{A}m\text{ ​}\left(\theta \right)+E_{M}\left[1-m\text{ ​}\left(\theta \right)\right]} \end{array}$$

and

(11) $$\begin{array}{l} \alpha_{f\text{​},par}\text{​​}\left(\theta \right)=\frac{m\text{ ​}\left(\theta \right)E_{M}\alpha_{M}+\left[1-m\text{ ​}\left(\theta \right)\right]E_{A}\alpha_{A}}{m\text{ ​}\left(\theta \right)E_{M}+\left[1-m\text{ ​}\left(\theta \right)\right]E_{A}}\\ E_{f\text{​},par}\text{​​}\left(\theta \right)=m\text{ ​}\left(\theta \right)E_{M}+\left[1-m\text{ ​}\left(\theta \right)\right]E_{A} \end{array}$$

Respectively (note that the mass densities of the two SMA stable phases are very close), and as far as the emerging phase (austenite on heating/martensite on cooling) occurs by random nucleation, a reasonable assumption is to take α*_f_*(*θ*) and E*_f_*(*θ*) as the average of the two bounding isostress and isostrain approximations.

When it comes to deciding which composition will be used for the Ti-Ni-Cu film, the fact that transformation temperatures above RT and also that a narrower hysteresis (as far as this does not affect the shape recovery to a great extent) are preferable were considered. Thus, Ti-Ni-Cu alloys with Cu content exceeding 10% (in the B2 ↔ B19 transformation range) have the advantage of higher transformation temperatures, but once the Cu content increases above 15%, the shape recovery is substantially diminished. Therefore, Cu content between 12.5% and 15% could be a suitable choice for Ti-Ni-Cu alloy-based microactuators design.

## 3. Results

Experiments were mainly focused on the Ti-Ni-Cu target, which is expected to exhibit low thermal hysteresis, and reasonably good shape recovery, below 15% at Cu content. The target was investigated before and following the DC magnetron sputtering process. The EDX analysis showed 52.28 at.% Ti, 33.34 at.% Ni, and 12.29 at.% Cu, thus locating the alloy in the compositional range where several phase transformations occur.

### 3.1. Target SMA Materials Characterization

Ti-Ni alloys are the most-used SMAs for film deposition, their properties being strongly influenced by the composition and thus by the temperature at which they are measured, i.e., if they are in the martensite or austenite states or in the transition temperature range, where the two phases coexist. Their thermal and elastic properties are summarized in Table 1. These properties are known in the literature for the austenite (B2) and monoclinic martensite (B19’) phases of the Ti-Ni alloys, whereas less information exists about the Ti-Ni-Cu alloys, especially about the orthorhombic B19 martensite.

**Figure 1:**
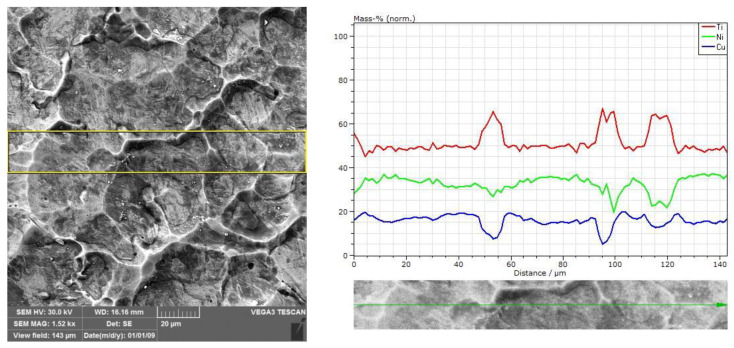
(**left**) Microstructure and (upper (**right**)) compositional map of the Ti-Ni-Cu target alloy; the investigated area is marked by (**left**) a rectangle and by (lower (**right**)) a double-ended arrow.

The microstructure of the Ti-Ni-Cu alloys is influenced by the composition, and since the replacement of Ni by Cu can be in a wider range, the segregation of secondary phases is not an uncommon consequence [46,47]. On the other hand, the transformation temperatures are more stable with respect to the Ni/Cu composition compared to Ni/Ti ratio in SMAs. The microstructure and composition of the Ti-Ni-Cu target are shown In Figure 1. It can be observed that polyhedral grains in the range of 40–60 µm are individualized by well-delimited grain boundaries.

Further compositional analysis along the surface indicates strong variation in the grain boundaries compared to the one determined inside the grains. Thus, an increase in the Ti content in the grain boundary and, as shown in Table 2, a decrease in the Cu and Ni content in the same area were detected. Whereas the composition inside the grain is closer to the overall composition of the target, in what concerns the grain boundaries, the situation is markedly different.

**Figure 2:**
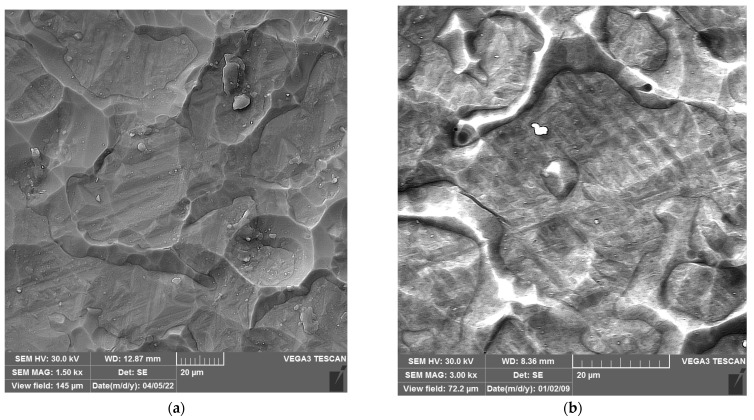
Surface SEM micrograph of the Ti-Ni-Cu target alloy indicating the presence of orthorhombic martensite inside the grains, as well as details about the grain boundaries and precipitates: (**a**) 1500× magnification SEM; (**b**) 3000× magnification.

In addition, one may observe (see Figure 2) that the martensitic structure is visible inside the grains and also that this is discontinued by the Ti-rich precipitates.

**Figure 3:**
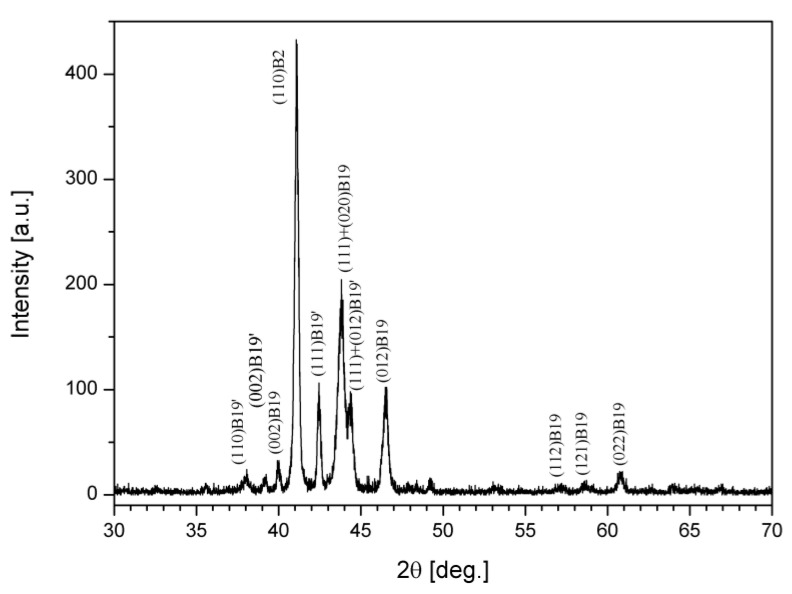
XRD record of the Ti-Ni-Cu target.

The X-ray diffraction record of the target, in Figure 3, shows the coexistence of the B2, B19 and B19’ phases, indicating the proximity of the two transformations, i.e., B2 → B19 and B19 → B19’ [20].

**Figure 4:**
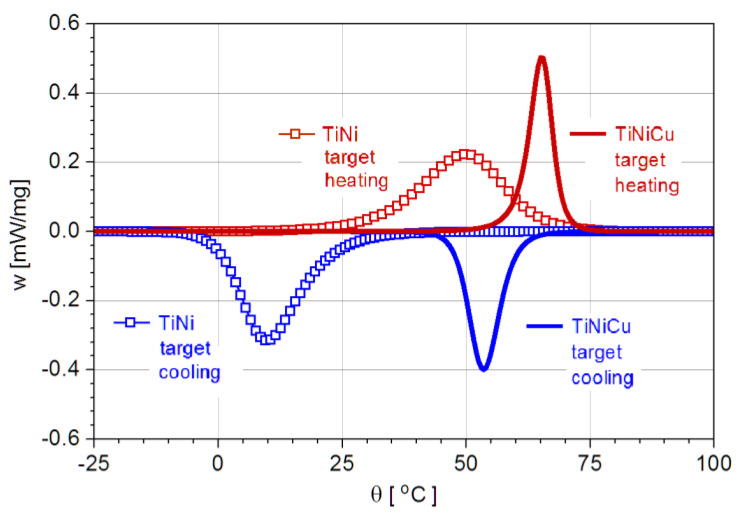
DSC records of the MPT in the target alloys used for experiments: Ti-Ni, showing the B2 → B19’ transition, and Ti-Ni-Cu, showing the B2 → B19 transition.

The microactuation in shape-memory alloy bimorphs can be predicted based on the analysis of the phase transformation in the shape-memory alloy for a given geometry of the actuator and a specific substrate, and the first step in this sense is to derive the dependence m(*θ*); in Figure 4, the DSC records of the two alloys used in experiments.

**Figure 5:**
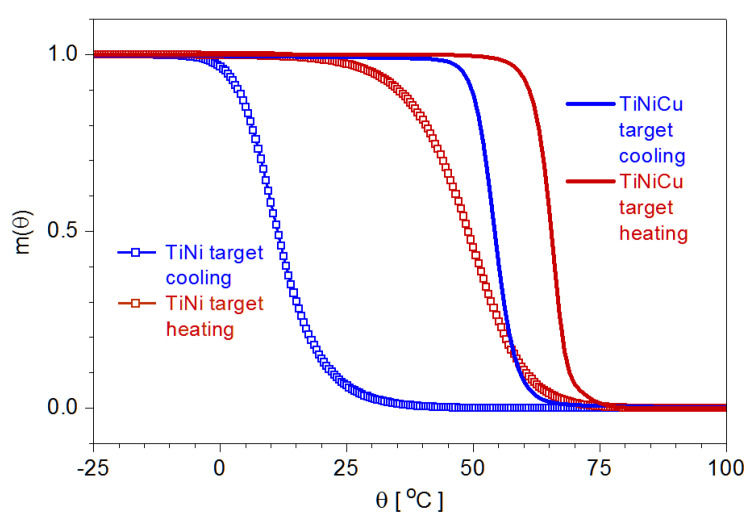
The thermal evolution of the martensite volume fraction for the target alloys.

By applying Equations (4) and (5) to the as-recorded DSC data, the temperature dependence of the martensite volume fraction was calculated for the two branches of the complete thermal cycle; in Figure 5, the results are plotted. Here, it can be seen that compared to the Ni-Ti alloy, the Ti-Ni-Cu exhibits a significantly narrower thermal hysteresis of the MPT.

The experiments were mainly focused on the Ti-Ni-Cu target, which is expected to exhibit low thermal hysteresis and reasonably good shape recovery, below 15% at Cu content. The target was investigated before and following the DC sputtering process. The EDX analysis of the composition showed 52.28 at.% Ti, 33.34 at.% Ni, and 12.29 at.% Cu, locating the alloy in the compositional range where several phase transformations occur. 

### 3.2. Sputter-Deposited Ti-Ni-Cu Films on Kapton Characterization

The analysis of the composition of the sputtered films compared to the one of the target—detailed in Table 3—shows a decrease in the Ti content and a corresponding increase in the Ni content close to the 50:35:15 ratio between the Ti and Cu elements in the range where the transformation sequence is B2 → B19 → B19’, with the Ms temperature around 60 °C, according to the analysis of the TiNiCu system performed by Myiazaki et al. [8].

**Figure 6:**
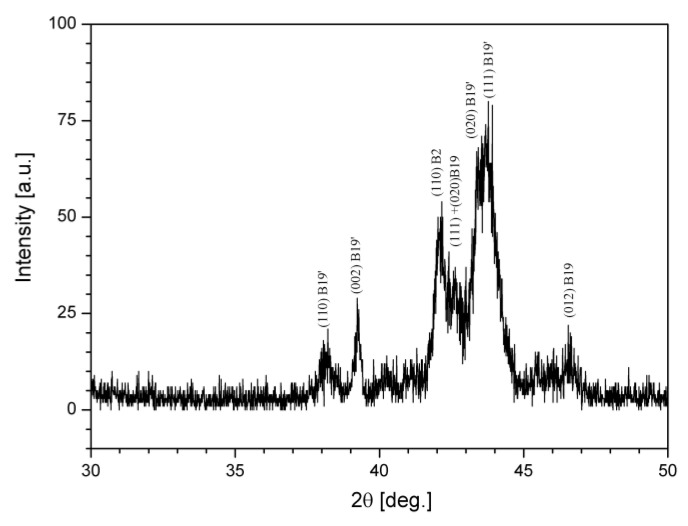
XRD record of the sputtered Ti-Ni-Cu film.

It was observed in our analysis that the films deposited at 550 °C showed full crystallization, and as it can be seen in the XRD record in Figure 6, B2, B19, and B19’ peaks coexist, which suggests a mixture of phases at RT.

**Figure 7:**
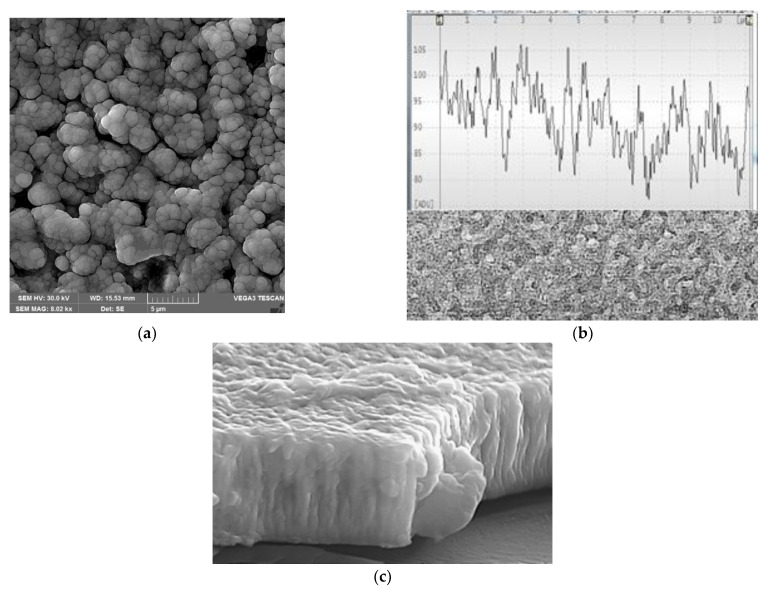
SEM images of the sputter-deposited Ti-Ni-Cu film: (**a**) surface image showing ultrafine microstructure; (**b**) surface topography analysis indicating micrometer and sub-micrometer grains; (**c**) cross-section image (8.600× magnification).

The SEM analysis of the microstructure of the sputtered NiTiCu films, presented in Figure 7, showed clusterization of ultra-fine grains of about 500 nm in the range of 2–5 µm. A fractured region of the film reflects a typical columnar growth as a packed structure growing perpendicular to the substrate. The lack of voids and the densely packed fibrous grains locate the film in the transitional range of the Thornton diagram [48], where a competitive texture starts to develop around half of the melting temperature.

**Figure 8:**
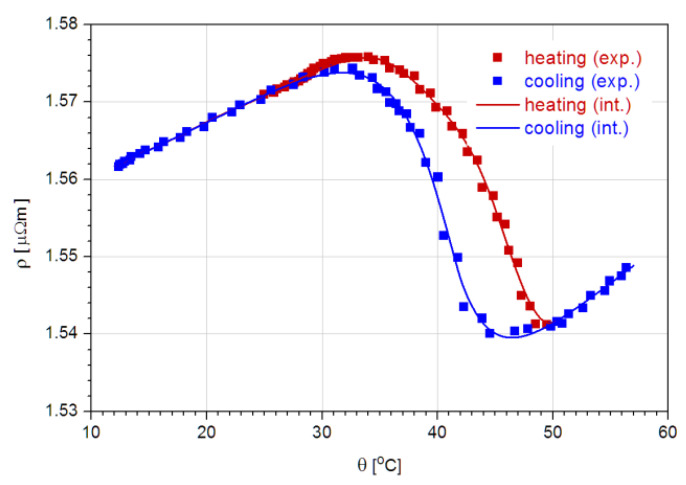
Evolution of the electrical resistivity of the Ti-Ni-Cu film along a heating/cooling cycle, which includes both the forward and reverse MPT branches (symbols: measured points, solid lines: interpolated data).

It is concluded that the films are crystalline, and thus they are expected to exhibit shape-memory properties and contribute to stress relief in the bimorph structure. This is further confirmed by the R(*θ*) measurements and ρ(*θ*) plot, showing the MPT (Figure 8).

According to Busch et al. [49], who analyzed the crystallization of 10µm-thick films deposited by DC magnetron sputtering on glass substrates, it was found that crystallized films showed transition temperatures that were lower than those of the parent material; thus, certain Ti loss is expected in the films. However, in the films deposited on Kapton in the present study, only slight differences were found between the DSC data of the target and the film. In fact, the martensitic phase transformation was distinctively observed during heating and cooling in the 25–115 °C range. According to the measured results using different techniques, the differences can be attributed to the accuracy of the measurement, especially to the control of the heating and cooling rate.

The thermal evolution of the Ti-Ni-Cu resistivity during the forward and reverse MPT was used for m(*θ*) determination. From the interpolated ρ(*θ*) data, numerical values of this volume fraction were obtained and were accurately fitted to the as-calculated data sets as superpositions of three transition functions (Logistic Dose Response in this case):

(12) $$\;m_{h}(\theta )=\frac{a_{\;h1}}{1+{\left(\frac{\theta }{b_{\;h1}}\right)}^{c_{\;h1}}}\;+\frac{a_{\;h2}}{1+{\left(\frac{\theta }{b_{\;h2}}\right)}^{c_{\;h2}}}+\;\frac{1-(a_{\;h1}+a_{\;h2})}{1+{\left(\frac{\theta }{b_{\;h3}}\right)}^{c_{\;h3}}}\;$$

and

(13) $$m_{c}(\theta )=\frac{a_{c1}}{1+{\left(\frac{\theta }{b_{c1}}\right)}^{c_{c1}}}\;+\frac{a_{c2}}{1+{\left(\frac{\theta }{b_{c2}}\right)}^{c_{c2}}}+\;\frac{1-(a_{c1}+a_{c2})}{1+{\left(\frac{\theta }{b_{c3}}\right)}^{c_{c3}}}\;$$

**Figure 9:**
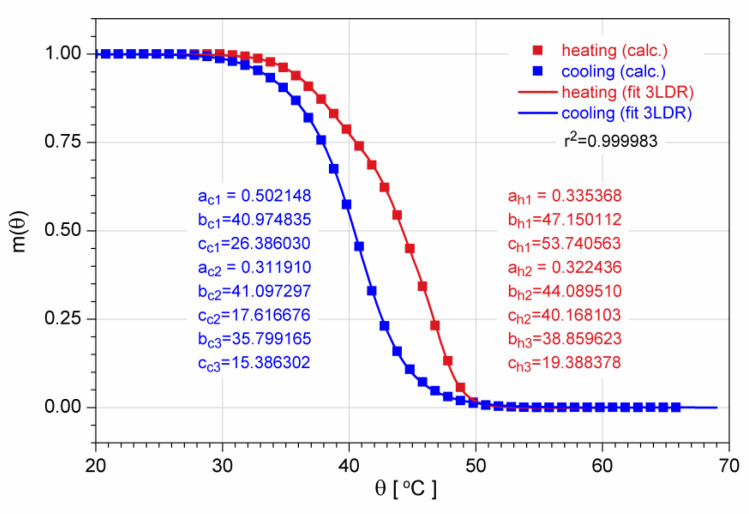
Temperature dependence of the martensite volume fraction of the Ti-Ni-Cu film along the forward and reverse thermal cycle, which includes the MPT (symbols: experiment-generated data, solid lines: eye guiding, fitting parameters, and coefficient of determination also included.

The high value, very close to unity (r^2^ = 0.999983), of the coefficient of determination confirms the goodness of the fit (subscripts h and c stand for heating and cooling, respectively). In Figure 9, the plots of the two functions are shown together with the data resulting from measurements.

### 3.3. Actuation; Experiment vs. Modeling

A sequence of effects take place as the temperature of the SMA-based bimorph is changed from the deposition temperature and further on during thermal cycling, i.e.:

− The thermoelastic stress on cooling from the deposition temperature first develops in the austenite phase of the SMA film;
− This stress leads to a bimetal effect, i.e., a curvature and a deflection of the free end of a bimorph cantilever, proportional to the difference between the deposition temperature and the instant temperature, respectively, occur;
− Once the MPT is initiated (at M_s_ temperature, on cooling) in the SMA film, the stress relief that occurs in the bimorph due to the formation of the softer martensite can also be observed as the unbending of the cantilever-type actuator;
− The actuation of the bimorph during the austenite → martensite transformation proceeds gradually, following the austenite/martensite ratio until the film is fully in the martensite phase (below the M_f_ temperature);
− Upon further cooling below the M_f_ temperature, the thermoelastic stress corresponds to the one that develops in the martensite film/substrate bimorph. The actuation slope as a function of temperature is less pronounced than the one with the film in the austenite phase;
− Upon heating, the actuation occurs in reverse order, during the martensitic transformation that leads to the gradual change from martensite into austenite in the A_s_–A_f_ temperature range, also associated with an increase in the stress in the bimorph and a change in the deflection of the free end of cantilever-type actuators.

**Figure 10:**
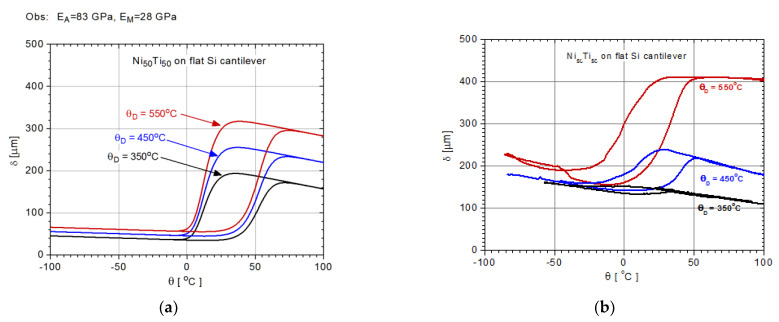
Deflection vs. temperature of the free end of similar bimorph cantilevers, with Ni–Ti films deposited on Si substrate at different temperatures (350 °C, 450 °C, and 550 °C): (**a**) model predictions; (**b**) experimental data.

With m(θ) defined, and for a specific composition of the target, the modeling of the actuation in the bimorphs with the SMA film deposited by sputtering depends on the deposition temperature and the geometry and thermomechanical properties of the film and the substrate. The relative thickness between the SMA film and the substrate is another key element. The deposition process frequently leads to SMA thicknesses in the range of 1–10 µm, while the thickness of the substrate varies in a larger range. Depositions on Si are made on substrates in the range of 100 µm, while the ones on polyimide substrate can vary in larger limits, usually from a few tens of micrometers to hundreds of micrometers. The larger the difference between the thermoelastic properties of the SMA film and the ones of the substrate, the higher the microactuation is expected for the same deposition temperature. In regards to the role of the deposition temperature, its expected effect on the bimorph actuation is an increase in the deflection, as illustrated in Figure 10 for one of the most studied bimorphs (TiNi/Si). Among the polymeric materials, Kapton (with the properties summarized in Table 1) is the most-used substrate due to its capacity to withstand high deposition temperatures (in the range of 500 °C). The analysis of the actuation in bimorph structure SMAs/polymer bimorphs was made using the model developed based on NiTi shape-memory alloys deposited on Si substrates [41] by replacing the substrate material properties with those of Kapton. The actuation models were determined for NiTi film thicknesses in the range of 1–10 µm and 25–150 µm for the Kapton substrate, taking a rectangular shape cantilever-type structure of 10 × 2 mm as a base.

An important element in modeling the phase transformation is film crystallinity. This is especially important because the films can be deposited at temperatures starting from RT to about 600–700 °C; the lower the deposition temperature, the less crystalline the SMA films are. Most of the modeling of the actuation assumed that the film was crystalline, regardless of the deposition temperature, and the models only focused on the amplitude of the deflection of the free end of the cantilever actuator. Thus, it becomes important to analyze the simultaneous effect of crystallinity and the deposition temperature on the resulting actuation of cantilever-type structures. The balance between the deposition temperature and the actuation is a key element, especially in what concerns the resulting actuation. A temperature that is too low does not favor the crystallization of the film; thus, the martensitic transformation. A temperature that is too high may affect the properties of the substrate (e.g., polyimide).

Using the m(*θ*) determined out of the DSC of the TiNi target, it was possible to create (via the model for actuation vs. temperature implementation) the actuation profile within the transformation range for depositions at different temperatures (Figure 10a). The results are compared with experimental data of the films deposited on Si substrates (Figure 10b).

It can be seen that the deposition temperature not only plays a key role in the actuation potential but also in the temperature range this actuation occurs. The lower the deposition temperature is, the larger the range of the actuation temperature and the smaller the deflection of the bimorph cantilever free end. In addition, a significant difference between the model prediction and experiment may occur in the case of films deposited at lower temperatures. Most likely, such films are partially crystalline, and apparently, the model does not take this fact into account. The film crystallization occurs in a wide temperature range, and partially crystalline films are characterized by a porous structure that evolves towards densely packed fibrous and columnar grains before a fully crystalline structure is formed. As such, the observed grain growth is also associated with a reduction in the grain boundaries that affect the martensite structure development. In addition, as the deposition temperature is increased, higher stress develops in the SMA film, and a stress-induced phase transformation is favored. 

The simulation was based on the m(*θ*) data extracted from the DSC record of the Ti-Ni target material, whereas the experimental actuation data were obtained using bimorph cantilevers with Ti-Ni films deposited by sputtering, a process during which slight compositional changes may occur, thus causing changes in the position and width of the MPT thermal hysteresis (Ti loss will shift the hysteresis towards lower temperatures [50]). However, in the case of partially crystalline films, provided the volume fraction of the amorphous phase and its elastic modulus and coefficient of thermal expansion are known, a better prediction may be obtained by rewriting Equations (9)–(11) for a biphasic or triphasic (outside or inside the MPT thermal range, respectively) composite. In the case of actuators for which the deposition is carried out on polymeric materials, in order to achieve maximum actuation, it is important to ensure the highest possible deposition temperature for which the substrate properties are still not altered.

**Figure 11:**
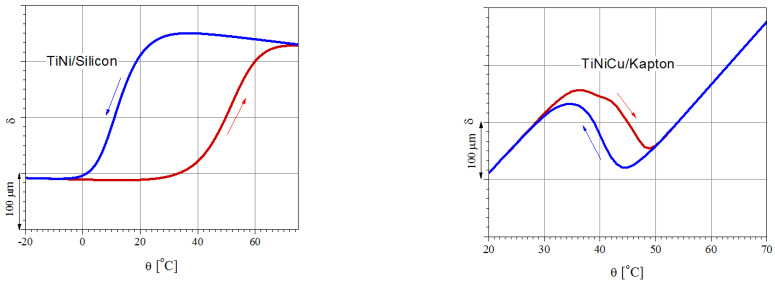
Actuation profile vs. temperature (deflection of the free end of bimorph cantilevers with NiTi and TiNiCu films deposited on different 100 µm-thick substrates, as predicted for 1 µm-thick film deposited at 550 °C and thermally cycled ((**left**): NiTi/Si; (**right**) NiTiCu/Kapton; arrows indicate heating and cooling, respectively).

In the fabrication of SMA-based bimorph actuators, besides the film composition, the influence of different thermoelastic properties of the substrate can play a key role in the resulting actuation. In the compositional range corresponding to the B2 → B19 MPT, the Ti-Ni-Cu SMAs are known to show a narrower hysteresis—as also observed in the DSC data—compared to the NiTi ones. On the other hand, the deflection curvature for the deposition on Kapton is in the opposite sense compared to the deposition on Si (Figure 11).

**Figure 12:**
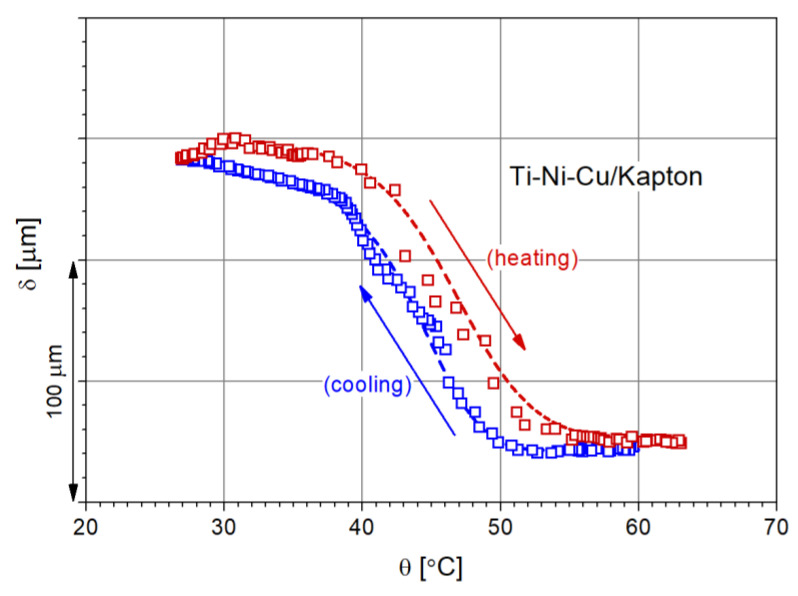
Deflection vs. temperature of the free end of a bimorph cantilever with Ti-Ni-Cu film deposited at 550 °C on Kapton (arrows indicate heating and cooling, respectively).

This is shown in Figure 12 where the laser measurements of the free end of the bimorph cantilever deflection are plotted vs. temperature for a bimorph with a Ti_52.28_Ni_33.3_Cu_12.29_ film deposited on Kapton.

The experimental results depicting the actuation of the bimorph are consistent with the model developed, and the shifting of the transformation temperatures with respect to the ones of the target is also linked to the compositional changes that occur when transferring the SMA from the target to the substrate during the magnetron sputtering process. It is technically more feasible to determine the transformation temperatures of the target using DSC measurements and to evaluate the potential actuation range of bimorphs using a model that would allow a first image of what can be expected.

## 4. Conclusions

The actuation in bimorph structures with SMA films on Si and Kapton substrates was analyzed based on the model that uses the fraction of martensite determined out of DSC thermograms of the targets used for sputtering. The comparison between the actuation using Ni-Ti and Ti-Ni-Cu SMA films reflects the specifics of the MPT, i.e., the B2 → B19’ transition for Ni-Ti and B2 → B19 for Ti-Ni-Cu, the latter one showing a reduced width hysteresis. In the model analyzed using the thermoelastic properties (thermal expansion coefficient; Young’s modulus) of Ti-Ni alloys in both the austenite and martensite states and those of the substrate and for a given geometry of the actuator, the bimorph with films deposited on Si showed a bending toward the film, whereas that with films deposited on Kapton showed a bending toward the substrate, but with a smaller amplitude.

The models were used to analyze the effect of the deposition temperature on the resulting actuation, and the results were compared with experimental data. Based on the differences between the model and the experimental data, it is concluded that the model does not correctly reflect the actuation for depositions at low temperatures that lead to films that are not crystalline. The crystallinity of the SMA films in the bimorph is a factor affecting the resulting actuation. In fact, the experimental results show a reduced actuation for films that are not crystalline. This was attributed to the particular microstructure of the partially crystalline films, where, according to the Thornton diagram, the grains were either not well individualized or the extended number of grain boundaries limited the effect of the martensitic transformation.

The experiments were focused on Ti-Ni-Cu shape-memory alloys in the compositional range where the B2 (monoclinic) → B19 (orthorhombic) phase transformation occurs. The target used for the magnetron sputtering of the films showed grains in the range of 40–60 µm, with Ti-rich boundaries and precipitates. Martensite was observed inside the grains, and its presence was confirmed by X-ray diffraction, together with the Ti-rich phases.

The sputtered Ti-Ni-Cu films on the substrate heated at 550 °C revealed that a loss of Ti occurred during the deposition leading to a composition of Ti_52.28_Ni_33.34_Cu_14.38_ (at.%). Based on the SEM characterization of the film, a clusterization of ultra-fine grains of about 500 nm in the range of 2–5 µm was observed, with a typical columnar growth as a packed structure growing perpendicular to the substrate.

The analysis of the actuation as a function of the temperature of bimorphs with Ti-Ni-Cu films deposited on the Kapton substrate using a laser sensor with the spot projected on the free end of the cantilever-type structure showed a deflection consistent with the predicted model in terms of the shape and orientation of the curvature and proving the relationship with the martensitic transformation in the film.

The measurement of the electric resistance of the films as a function of temperature confirmed the martensitic transformation and the possibility to be used to generate the fraction of martensite vs. temperature data, which has the potential to be further used to model the microactuation of bimorphs with SMA film.

## Figures and Tables

**Table 1:** Selected thermal and mechanical properties used in the model for the actuation study.

SMA Film/Substrate		Young’s Modulus, E_A_ (GPa)	Coefficient of Linear Expansion, *α* (10^−6^/C)
NiTi	B2 austenite	60–85	11/°C
	B19‘ martensite	20–50	6.6/°C
Ti_50_Ni_30.2_Cu_19.8_ [44]	B2 austenite	58	-
	B19 martensite	28.5	-
Silicon		130	2.67/°C
Kapton^®^ 100HN [45]		2.76	20/°C

**Table 2:** Compositional analysis of the Ti-Ni-Cu target.

Element	Ti		Ni		Cu	
	at.%	wt.%	at.%	wt.%	at.%	wt.%
Overall	59.32	53.65	26.77	29.64	13.91	16.71
Grain	54.18	48.51	32.5	35.66	13.32	15.83
Boundary	68.89	62.77	13.96	16.37	17.15	20.86
Precipitate	68.38	62.81	14.44	16.25	17.18	20.94

Error (1Sigma) in the range of 0.41 to 1.70 [wt%].

**Table 3:** Comparative compositional analysis of the Ti-Ni-Cu target and film.

Element	Ti		Ni		Cu	
	at.%	wt.%	at.%	wt.%	at.%	wt.%
Target	59.32	53.65	26.77	29.64	13.91	16.71
Film	52.28	46.58	33.34	36.41	14.38	17.01
